# Water evaporation as a function of temperature, humidity, air velocity and body size in inactive terrestrial pulmonate *Theba pisana*

**DOI:** 10.1186/s40850-025-00236-0

**Published:** 2025-07-07

**Authors:** Sascha Zimmermann, Ulrich Gärtner, Yvan Capowiez, Heinz-R. Köhler, David Wharam

**Affiliations:** 1 Mesoscopic Physics and Nanostructures, Institute of Applied Physics, University of Tübingen, Auf der Morgenstelle 10, D-72076 Tübingen, Germany; 2 Animal Physiological Ecology, Institute of Evolution and Ecology, University of Tübingen, Auf der Morgenstelle 5, D-72076 Tübingen, Germany; 3 INRAE, UMR1114, Unite EMMAH, Site Agroparc, Avignon, Cedex 09 F-84914 France

**Keywords:** Evaporation, Pulmonate, *Theba pisana*, Thermodynamics, Water loss, Temperature, Relative humidity, Air velocity

## Abstract

The conservation of water is of great importance to terrestrial snails in order to survive in arid environments. However, evaporation of body water does occur, and it is unclear whether snails are able to actively reduce the evaporation or whether increased environmental temperature inevitably leads to increased evaporation. Physically, the quantity of evaporating water is largely determined by the temperature, the relative humidity and the velocity of the surrounding air, as well as the surface area of the evaporating body. *Theba pisana*, a widespread Mediterranean land snail, is exposed to a wide range of ambient temperatures. For this species the experimentally recorded evaporation rate per mass was tested as a ‘response’ variable to the ‘explanatory’ variables temperature, relative humidity and air velocity using multiple regression modelling. The variation in specific evaporation of *T. pisana* could be explained to 72.8% by these parameters. Threshold temperatures, which depended on the parameters relative humidity, air velocity and body size, were found to induce an active response in the snail that reduced their evaporation rate. A comparative analysis of the specific evaporation rate of different-sized snails shows that, at low temperatures, larger snails have a lower specific evaporation rate than smaller snails. However, above a threshold temperature this trend reversed, depending on air velocity and relative humidity. In general, the evaporation rate is lower at higher relative humidities. In the specified temperature range (23–35 °C) the snails tend to actively reduce the evaporation rate to conserve water rather than increase the evaporation rate to avoid overheating. These findings provide valuable information about the physiological adaptations that support survival in water-limited environments, offering important implications for understanding arid-adapted species such as desert snails.

**Supplementary Information:**

The online version contains supplementary material available at 10.1186/s40850-025-00236-0.

## Introduction

The land snail species *Theba pisana* (Müller, 1774), belonging to the family Helicidae within the suborder Eupulmonata, first appeared in the Mediterranean and has since extended its habitat along the Atlantic coast, stretching from Morocco to Ireland [1]. The life cycle of this bush-dwelling species can either be annual or biennial, and largely depends on the conditions of its habitat [2]. The snails examined in this study most likely have an annual life cycle, as they came from the northern Mediterranean, which has a habitat at the warmer end of the range for this species [3]. The annual life cycle is as follows: reproduction of this species occurs between autumn and winter [3], the juveniles hatch in winter, grow in spring and reach their adult size by summer [4].

Given that the water content of eupulmonates is usually high, for instance, exceeding 75% in the related species *Cantareus apertus* [5], it is crucial for them to avoid extreme water loss, even though snails can survive with a loss exceeding 35% of their water content [6]. Dehydrated snails take water in through the body surface, by drinking and by feeding, although the latter seems to be of limited significance to the water balance of terrestrial pulmonates [7–9]. Dehydrated *Otala lactea* and *Helix aspersa* moving over a wet surface reach a full hydrated state within 2–3 h [10]. The water uptake of *Helix aspersa* through the skin in saturated air is about 5% of the body mass per week [11].

A hot environment therefore imposes significant thermal stress on terrestrial snails and, in extreme cases, dehydration can lead to premature death. In order to prevent dehydration or excessive water loss while searching for food, these snails exhibit most activity during periods of high humidity or during the night [12]. During daytime both juvenile and adult snails tend to avoid areas of excessive heat and direct sunlight, such as heated soil surfaces. In general, the activity of gastropods decreases with increasing temperatures [13]. They also exhibit a sensitivity to the wind direction [14] and, consequently, when they climb vertically oriented objects they adjust their orientation in order to find more favourable conditions. Doing so, these animals can remain in the burning sun at some distance from the ground in arid areas for weeks in an inactive state, or even months in the state of aestivation [15]. In such a state, water loss is minimal: for example, only one in six *Codringtonia* species showed any tendency to lose water at all in the experiments of Kotsakiozi et al. [16], and the water content of *Cantareus apertus* was only 14% lower (75% vs. 61%) compared to non-aestivating (active) snails after six months [5]. The disadvantage of aestivation is that the snails cannot respond to excessive body temperatures with evaporative cooling [17]. Very high temperatures (40–42 °C) can induce flight, and combinations of high humidities and lower temperatures, which allow the search for food, induce *T. pisana* arousal of aestivation [17, 18].

Most of the water loss occurs through the aperture of the snail’s shell, although there is some evaporation through the shell itself [6]. Water loss through the shell is less than 5% of the water loss measured through the aperture in *Cepaea nemoralis*, *Cepaea hortensis* and *Arianta arbustorum* [19]. To prevent water loss through the aperture, snails tend to seal the shell aperture directly to the substratum [6] with mucus, which can be enriched with calcium carbonate to form a so-called epiphragm. Compared to the water loss through the aperture, the water loss with an epiphragm in *Helix aspersa*, *Otala lactea*, and *Sphincterochila zonata* could be reduced by about 30–40% [6]. Even if no epiphragm is formed when a terrestrial snail becomes inactive and withdraws into its shell, it leaves an exposed surface. However, the evaporation rate still decreases rapidly within 20–40 min [20]. *Otala lactea* can maintain low rates of water loss while being inactive for at least several weeks over a wide range of relative humidities (1.5% to near saturation) compared to the water loss of active snails [21]. The water loss through the skin of an active, outstretched *Helix aspersa* is similar to that from a free water surface [22].

The fundamental physical basis for the phenomenon of evaporation is the difference between the saturation pressure and the generally lower partial pressure of water in the air, resulting in a pressure gradient between the aqueous environment inside the snail and the surrounding air. This allows the air to absorb further moisture, which is released by the snail. The presence of wind serves to increase the rate of evaporation by transporting water molecules away from the snail. Consequently, the saturation deficit and the air velocity should have an effect on the evaporation rate.

The impact of thermal stress on terrestrial snails in their environment and their reaction to the stress factors have been thoroughly studied by Schweizer et al. [17]. In order to model the impact of different heat sources on the thermal status of a single individual, a numerical simulation of the heat balance has recently been carried out based on the thermodynamic interactions between the animals and their environment [23]. The different parameters taken into account were solar and terrestrial radiation along with the radiation of the snail itself, evaporation, metabolism, and convection. The model also incorporates the snail’s intrinsic parameters, which are the size of the individual and its height above the ground.

While we have a basic understanding of the theoretical connections between these crucial thermodynamic parameters and the snail, our comprehension of their quantitative relationships is still quite limited. Initial simulations run by this model, using approximate numerical values for a hot Mediterranean summer day and two distinct snail body sizes at two varying elevations from the ground, indicated that evaporation from a land snail’s body could contribute for up to about 4% of the total potential for body cooling throughout the day and of about 11% during the time of day when body temperatures are highest. Although these values seem quite low, the water loss over an extended period of time can still lead to serious dehydration. Based on this assumption, land snails should attempt to reduce their water loss strongly as temperatures approach their tolerance limit.

In the present study, we therefore investigated whether terrestrial snails are able to actively reduce their evaporation and thus avoid dehydration, or whether increased ambient, and thus also body temperature leads to an increased evaporation and hence avoids overheating. The measurement was conducted at increased temperatures, considering the entire temperature range of the habitat.

We also will relate our findings to previous studies on evaporation in terrestrial snails. The water loss of various pulmonate species has been studied in relation to several affecting factors, and numerous studies have already documented the impact of temperature on evaporation in terrestrial snails [7, 19, 24–31], and we relate our findings to previous studies on evaporation in terrestrial snails.

## Materials and methods

### Test animals & experimental design

**Fig. 1:**
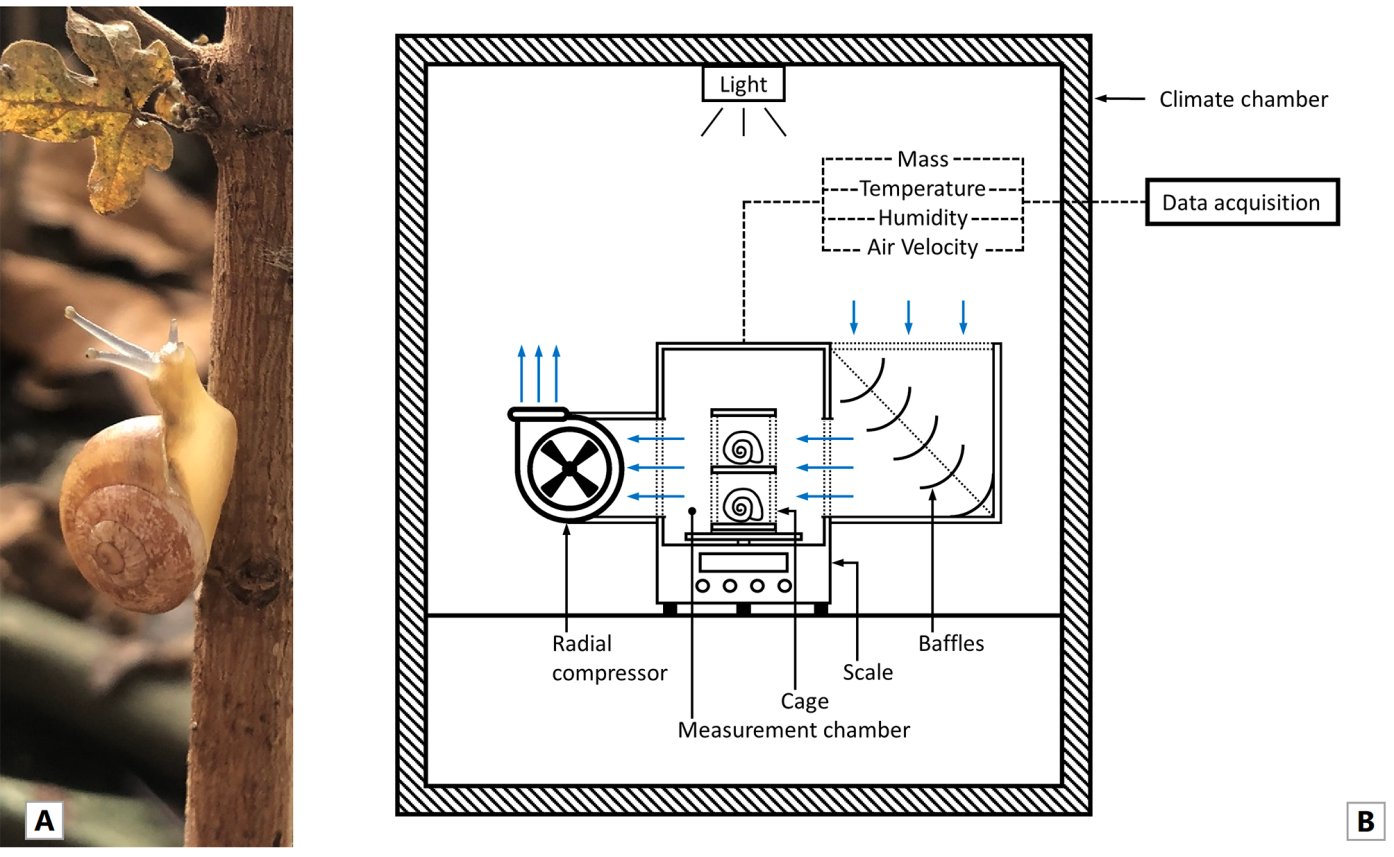
(**A**) An individual of *T. pisana* collected in the Avignon area of Southern France. (**B**) Schematic structure of the evaporation measurement system. Explanation of all details in the text

Individuals of the species *T. pisana* (Fig. 1A) were sampled from a grassland site on June 16, 2024 near Montfavet in the Avignon area of Southern France (43° 54.984′N, 4° 53.772′E) and subsequently shipped to Germany. The animals were kept at room temperature and natural light in ten transparent boxes (dimensions: 180 mm × 190 mm × 180 mm) with small ventilation holes. Damp paper towels were placed in these containers to maintain a humid environment. The animals were acclimated to these conditions for two weeks. The snails were checked daily, and dead individuals were removed. Twice a week the snails were fed lettuce and carrots, and the paper towels were replaced. To provide a natural source of calcium for the growth of the shells, a piece of cuttlebone was added to the boxes. The shell diameter (*d*), defined as the width of the shell from the opening coil to the opposite coil [32], hereafter diameter, was determined for each individual using a digital calliper (resolution 0.01 mm, accuracy ± 0.02 mm). For each evaporation measurement, a group of twelve individuals (*n* = 12) was randomly taken out of four boxes from the entirety. To ensure that the tested snails had time to recover, subsequent to each measurement, all snails were placed in the same box. The individuals for the next measurements were then taken from different boxes. The only selection criterion was the need for an almost identical individual size (*d*) within the group, as this allowed the calculation of evaporation rates on an individual basis. The mass (*m*, wet weight) of a group was determined with a precision scale (resolution 0.1 mg, accuracy ± 0.1 mg) immediately before each measurement. Each measurement started in the morning at approximately 8 a.m.

Although no specific legislation regulates experimentation with these organisms in Germany, all procedures involving snails were carried out in accordance with recognized best practices for invertebrate care and handling. We ensured that all efforts were made to minimize stress and harm to the animals, thus adhering to general animal welfare guidelines beyond legal requirements. To minimise disturbance to the animals before and during the measurements, they were kept in groups at a constant moderate temperature (20 °C), with a natural day-night rhythm, and fed food from their natural habitat.

### Experimental setup & quantification of the evaporation

To measure the water evaporation of a single individual over time at different temperatures, relative humidities and air velocities – the so-called environmental boundary conditions (BCs) – the snails were placed on a precision scale in a climate chamber (temperature range 0 to 65 °C, temperature accuracy ± 0.5 °C, relative humidity range 40 to 95%, relative humidity accuracy ± 5%). The accurate functioning of the chamber was regularly checked with an additional humidity sensor (temperature accuracy ± 0.5 °C, relative humidity accuracy ± 3%). The experimental setup is shown in Fig. 1B. Air velocity inside the measurement chamber was controlled by a radial compressor, and baffles ensured a directed airflow through the measurement chamber. The air velocity inside the climate chamber was measured with a hot-wire probe (velocity range 0 to 50 m × s^−1^, temperature range −50 to 80 °C, velocity accuracy 0.1 m × s^−1^ ± 0.5%). To prevent the snails from leaving the scale, each individual was placed in a small section of an air-permeable cage. Each section was small enough to ensure that the snails could barely move, but large enough to guarantee their attachment to the bottom or top of the chamber with their shell openings sealed with mucus.

After the snails were placed in the cage, the entire setup was weighed again and the mass of the cage subtracted in order to get an accurate starting mass. A single measurement of the evaporation lasted around 24 h. The first one hour of the measurements was not considered for the calculation of the evaporation due to the time required for the climate chamber to reach a stable state and for the snails in the cage to become inactive. This will be referred to as ‘run-in phase’.

We carried out 32 measurements in four test series at 23 °C, 27 °C, 31 °C and 35 °C. In each test series we tested two different relative humidity levels (*RH* ≈ 40% or 70%), two size classes (small or large) and two air velocities (*v*_*air*_ ≈ 0 m × s^−1^ or 1.2 m × s^−1^). The size class ‘small’ (*S*) included the snails with 12–14 mm and the size class ‘large’ (*L*) those with 16–18 mm diameter. There were no outliers in the size (diameter) of the respective group (*n* = 12). This allowed us to calculate the average mass loss for an individual by dividing the measured total mass loss the by the number of individuals in the group over time. It was assumed that the measured average mass loss over time was entirely due to the evaporation of the snails and is therefore referred to as evaporation rate $\dot{E}$ in mg × s^−1^ in the following, without the prefixed average.

The maximum standard deviation (*σ*) in temperature, relative humidity and air velocity, taking all measurements into account, was: *σ*_*ϑ,max.*_ = 0.06 K, *σ*_*RH, max.*_ = 3.32%/1.24% (*RH* ≈ 40%/70%), and *σ*_*v, max.*_ = 0.30 m × s^−1^, respectively.

### Mass and shell aperture size of the snails and their effect on the evaporation rate

Since the evaporation rate $\dot{E}$ does not indicate the size of the snail, and measures for snails of different sizes should not be compared directly, it is useful to give the specific evaporation rate $\dot{e}$. Relevant parameters values are the area of the shell aperture *A*_*sa*_, as most of the evaporation occurs through it, and the soft body weight of snail, the so-called shell-free mass *m*_*sf*_.

Twelve micro-computer tomography scans on *T. pisana* individuals in the size range of 9.9–18.6 mm diameter were previously performed by Zimmermann et al. [33]. To determine the area of the shell aperture, the scans were post-processed in ORS Dragonfly 2022.1.0.1259 (Object Research Systems (ORS) Inc., Montréal, QC, USA). The shell-free mass was derived from the wet weight of the living snail´s mass (*m*) by the following linear correlation, derived from the aforementioned data set (*R*² = 0.971):

1 $${m}_{sf}\left(m\right)=0.8026\times m$$

### Saturation deficit as physical principle of evaporation

The saturation deficit Δ*p*_*s*_ is defined as the difference between the saturation vapour pressure *p*_*v,s*_ and the actual vapour pressure *p*_*v*_. The saturation vapour pressure can be approximated using the Magnus equation [34], and the saturation deficit can then be calculated as follows

2 $$\eqalign{\Delta {p_s}\left( {\vartheta \>,RH} \right) =  &\  {p_{v,s}} \times \>\left( {1 - {{RH} \over {100}}} \right) \cr & = {C_1} \times \>{e^{{{{C_2}\> \times \>\vartheta \>} \over {{C_3}\> + \>\vartheta \>}}}} \times \>\left( {1 - {{RH} \over {100}}} \right) \cr} $$

with the constants *C*_*1*_ = 610.8 Pa, *C*_*2*_ = 17.08, *C*_*3*_ = 234.18 °C, and RH in % and ϑ in °C.

### Statistics

Due to the simple interpretation of the findings and the ability to analyse the relationship between a response variable and several independent explanatory variables simultaneously, multiple regression modelling (MLR) was given preference over other statistical approaches. ‘Specific evaporation rate’ as a response variable was modelled by MLR using the explanatory variables, ‘average temperature’, ‘average relative humidity’ and ‘average air velocity’. MLR was conducted with a standard least square fitting of the model. For MLR, the level of significance was set to ‘significant’ (*) for 0.01 < *p* ≤ 0.05, and to ‘highly significant’ (**) for *p* ≤ 0.01. In order to determine the absence of multicollinearity among the explanatory variables, variance inflation factors (VIF) were examined, with a resultant VIF < 5. The independence of the residuals was checked using a Durbin-Watson test, and the residuals were visually analysed for homoscedasticity and normal distribution. All of the aforementioned tests were performed in JMP 17.0 (SAS Institute Inc.).

The correlation of shell diameter and shell aperture area was determined with a fitted quadratic function in TableCurve 2D Version 5.01 (SYSTAT Software Inc.). All *p* and *R*^2^ values were calculated in JMP 17.0 (SAS Institute Inc.).

## Results

All relevant measurement data are listed in Table S1 in the Supplementary Materials.

### Shell aperture as a function of the diameter

On the basis of the CT scans (*n* = 12), a quadratic relationship between the diameter *d* and the area of the shell aperture *A*_*sa*_ of *T. pisana* was obtained (*R*² = 0.964):

3 $${A}_{sha}\left(d\right)=0.1805\times {d}^{2}$$

### Evaporation & specific evaporation

**Fig. 2:**
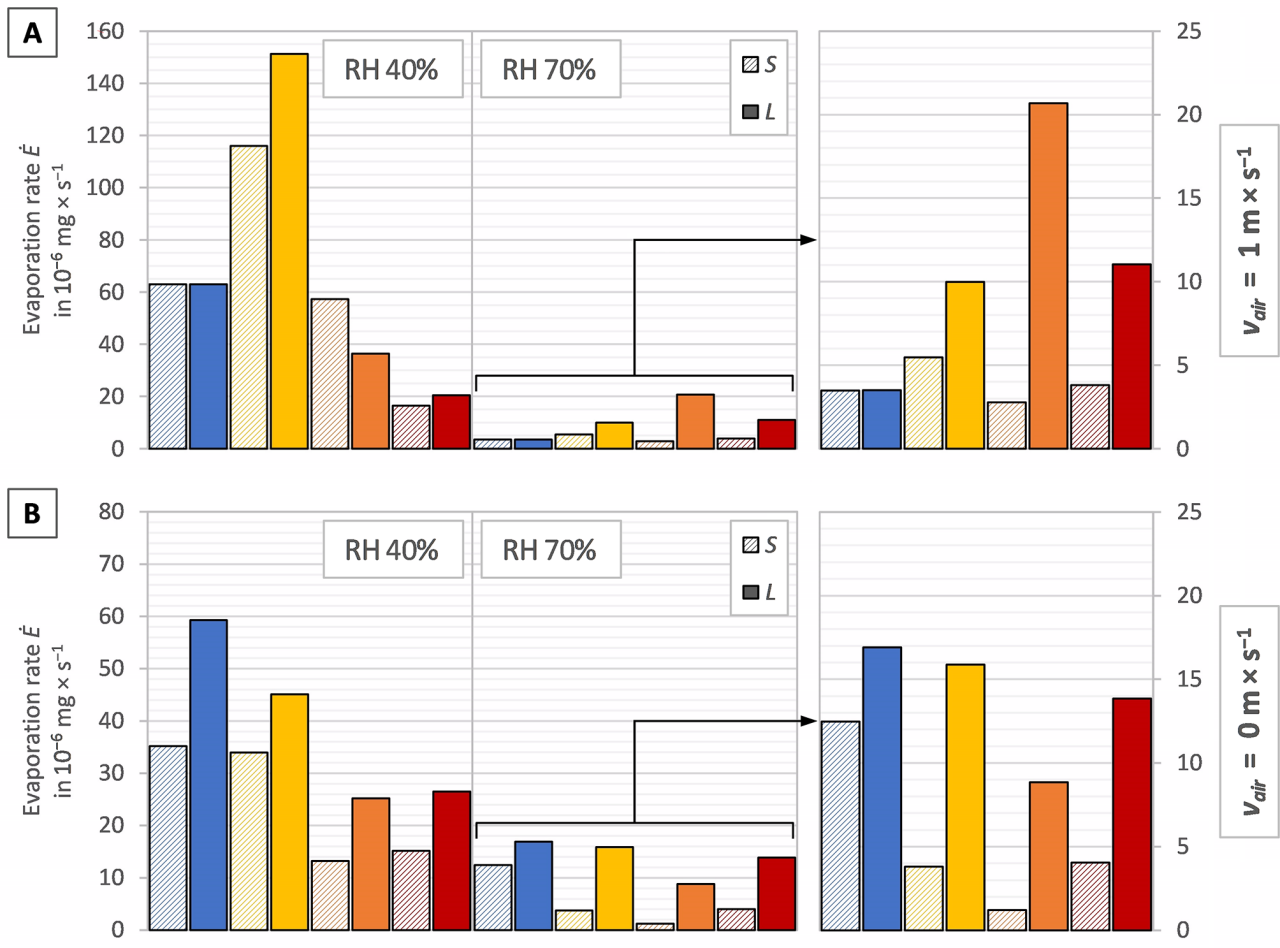
Evaporation rate $(\dot{E})$ with (**A**) a directed airflow (*v*_air_ ≈ 1.2 m × s^−1^) and (**B**) no directed airflow (*v*_air_ ≈ 0 m × s^−1^), in the different test series 23 °C (blue bars), 27 °C (yellow bars), 31 °C (orange bars) and 35 °C (red bars), with the two size groups *S* (left bar, hatched) and *L* (right bar, filled) of each test series, separated in the two relative humidities (40% left-hand side; 70% right-hand side)

**Fig. 3:**
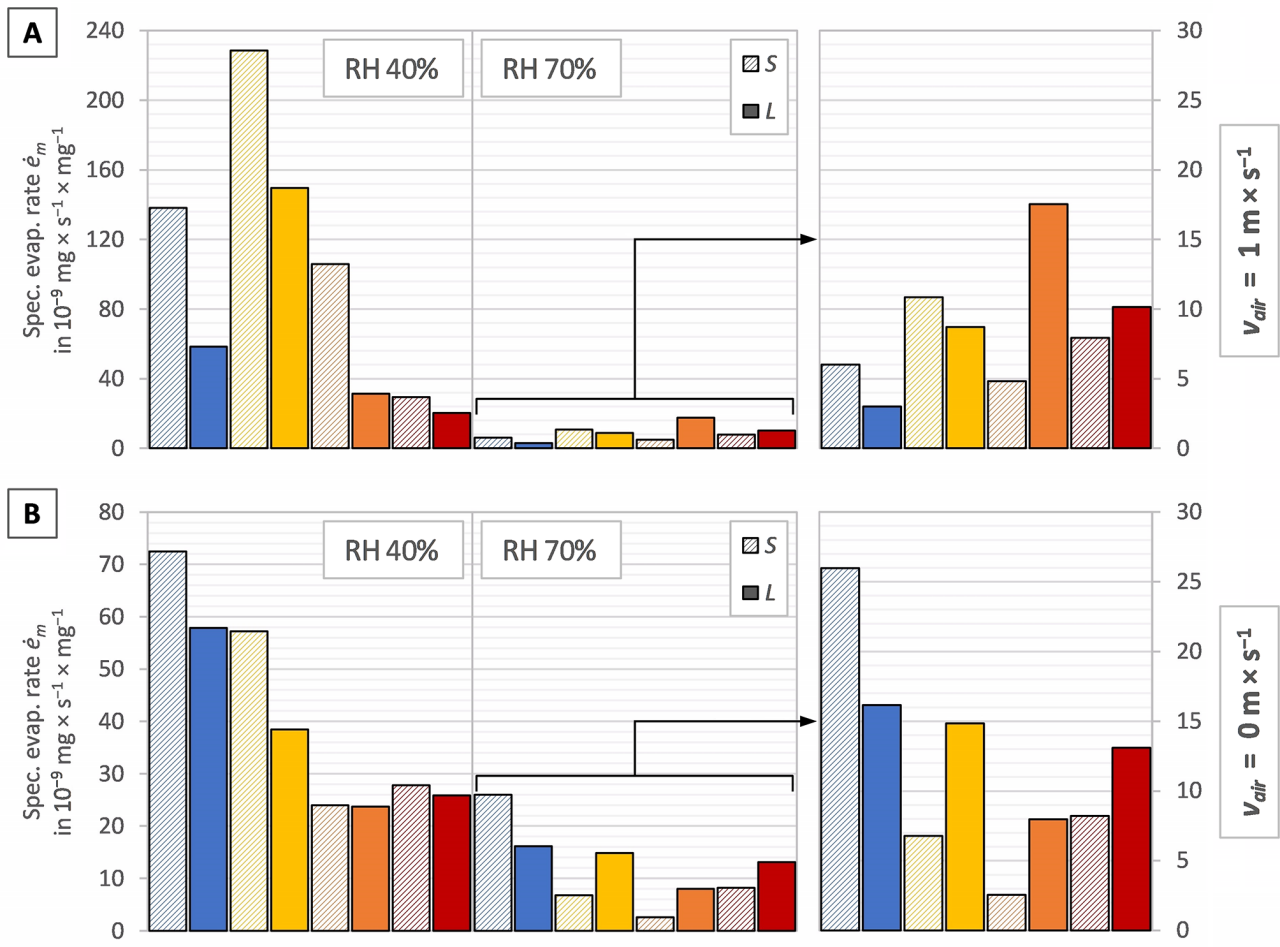
Specific evaporation rate per shell-free mass (${\dot{e}}_{m}$) with (**A**) a directed airflow (*v*_air_ ≈ 1.2 m × s^−1^) and (**B**) no directed airflow (*v*_air_ ≈ 0 m × s^−1^), in the different test series 23 °C (blue bars), 27 °C (yellow bars), 31 °C (orange bars) and 35 °C (red bars), with the two size groups *S* (left bar, hatched) and *L* (right bar, filled) of each test series, separated in the two relative humidities (40% left-hand side; 70% right-hand side)

**Fig. 4:**
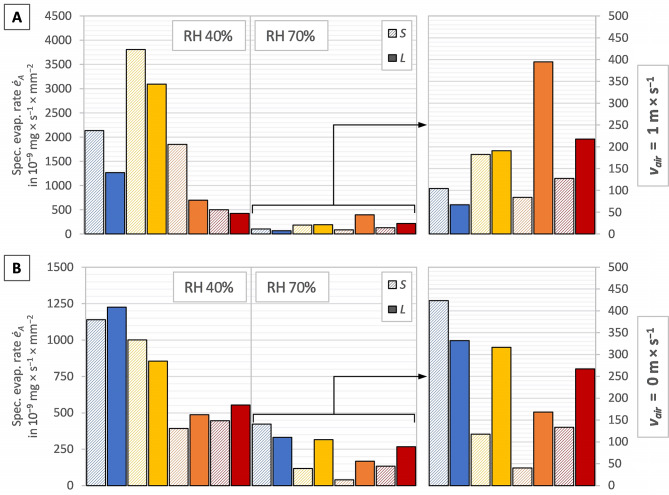
Specific evaporation rate per shell aperture area $({\dot{e}}_{A})$ with (**A**) a directed airflow (*v*_air_ ≈ 1.2 m × s^−1^) and (**B**) no directed airflow (*v*_air_ ≈ 0 m × s^−1^), in the different test series 23 °C (blue bars), 27 °C (yellow bars), 31 °C (orange bars) and 35 °C (red bars), with the two size groups *S* (left bar, hatched) and *L* (right bar, filled) of each test series, separated in the two relative humidities (40% left-hand side; 70% right-hand side)

The column graphs in Fig. 2A–B display the average evaporation rate $\dot{E}$, the column graphs in Fig. 3A–B display the average specific evaporation rate per shell-free mass ${\dot{e}}_{m}$, and the column graphs in Fig. 4A–B display the average specific evaporation rate per shell aperture area ${\dot{e}}_{A}$, per snail of the investigated snail groups in the test series 23 °C, 27 °C, 31 °C and 35 °C. Figures 2A, 3A and 4A display the measurements with a directed airflow of approximately 1.2 m × s^−1^, whereas Figs. 2B, 3B and 4B display the measurements with no directed airflow (*v*_*air*_ ≈ 0 m × s^−1^). Given that the values of the 70% measurements are comparatively low, these are displayed on a smaller scale on the right-hand side of the respective graph.

The evaporation rate, in general, was lower for the smaller snail groups in 29 out of 32 measurements. In two measurements $\dot{E}$ of the smaller snail groups and the larger snail groups were comparable (Fig. 2A, 23 °C at 40% & 70% RH) and in one measurement $\dot{E}$ was even higher for the smaller snail group (Fig. 2A, 31 °C at 40% RH). The three measurements that deviated from the general trend were those taken at an air velocity of 1.2 m × s^−1^ (Fig. 2A). The highest evaporation rate with a directed airflow (Fig. 2A) was observed at 27 °C at 40% RH in both groups. At 70% RH, the highest evaporation rate was observed at 27 °C in the “small” groups and at 31 °C in the “large” group. The highest evaporation rate with no directed airflow (Fig. 2B) was observed at 23 °C at 40% and 70% RH in both size groups.

Figure 3 illustrates, that smaller snails had a higher specific evaporation rate per shell-free mass ${\dot{e}}_{m}$ than larger individuals exposed to the same conditions at lower temperatures which changes with increasing temperature at a certain point. With a directed airflow (Fig. 3A) at 40% RH all smaller snail groups showed a higher evaporation rate and, at 70% RH, this changed between 27 °C and 31 °C. Without a directed airflow (Fig. 3B) at 40% RH, all smaller snails showed a higher evaporation rate, despite the values of 31 °C being almost identical and, at 70% RH, this changed between 23 °C and 31 °C. The highest evaporation rate with a directed airflow (Fig. 3A) was observed at 27 °C at 40% RH in both size groups. At 70% RH, the highest evaporation rate was observed at 27 °C in the small individuals and at 31 °C in the large individuals. The highest evaporation rate with no directed airflow (Fig. 2B) was observed at 23 °C at 40% and 70% RH in both size groups.

Figure 4 partially supports the trend displayed in Fig. 3: the smaller snails had a higher specific evaporation rate per shell-free mass ${\dot{e}}_{m}$ than the larger snails at lower temperatures which changes with rising temperatures at a certain point. With a directed airflow (Fig. 4A) at 40% RH all smaller snails showed a higher evaporation rate and, at 70% RH, this changes between 23 °C and 27, despite the values of 27 °C being almost identical. Without a directed airflow (Fig. 4B) at 40% RH, all smaller snails showed a lower evaporation rate, except for the data recorded at 27 °C. At 70% RH the value obtained for the smaller snails was higher at 23 °C, this changes at 27 °C and higher temperatures.

The highest evaporation rate with a directed airflow (Fig. 4A) was observed at 27 °C at 40% RH in both groups. At 70% RH, the highest evaporation rate was observed at 27 °C in the small individuals and at 31 °C in the large individuals. The highest evaporation rate with now directed airflow (Fig. 4B) was observed at 23 °C at 40% and 70% RH in both size groups.

**Fig. 5:**
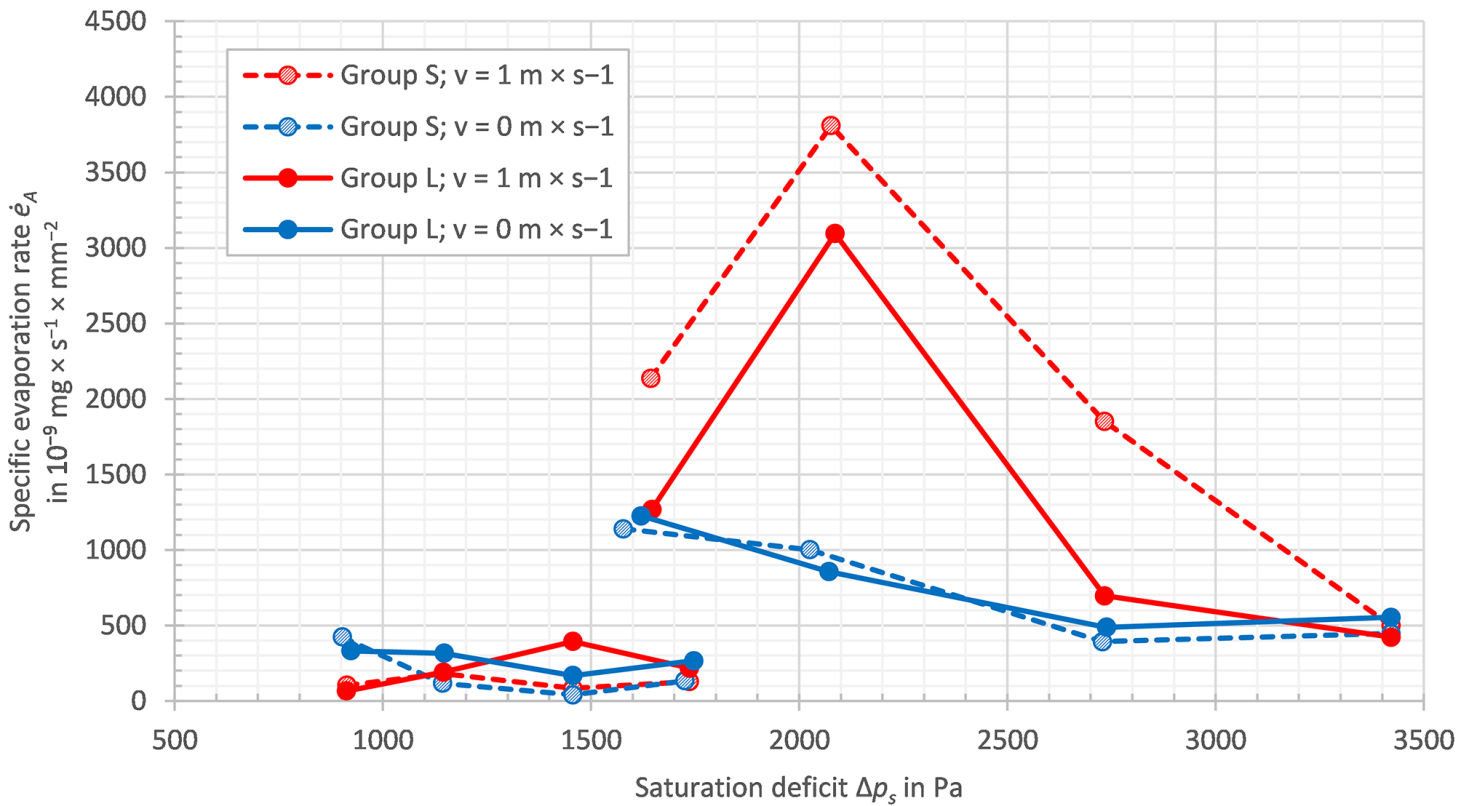
Specific evaporation rate per shell aperture area (${\dot{e}}_{A}$) vs. saturation deficit (Δ*p*_*s*_). Air velocities (red: *v*_air_ ≈ 1.2 m × s^−1^; blue: *v*_air_ ≈ 0 m × s^−1^) in the different size groups *S* (dashed lines, hatched circles) and *L* (solid lines, filled circles). The 16 data points corresponding to the 70% RH measurements are displayed on the bottom left of the graph, while the 16 data points corresponding to the 40% RH measurements are displayed on the upper and right side of the graph

The specific evaporation rate per shell aperture $({\dot{e}}_{A})$ is shown as a function of the saturation deficit (Δ*p*_*s*_) in Fig. 5. The data presented in this graph are represented in two distinct colours for the air velocities: red for measurements taken with no directed airflow, and blue for those with directed airflow. Dashed lines and hatched circles illustrate the size group *S* (small snails), whereas solid lines and filled circles illustrate the size group *L* (large snails). The data points on the bottom left of the graph refer to all the measurements conducted at 70% RH and the data points on the upper and right side of the graph refer to those conducted at 40% RH. All 32 data points are clustered (vertically) in eight different saturation deficits. The four data points within each cluster on the left-hand side of the respective relative humidity represent the test series 23 °C. The second cluster from the left of the respective RH represents the test series 27 °C, the third cluster from the left of the respective RH represent the test series 31 °C, and the cluster on the right-hand side of the respective RH represents the test series 35 °C. This indicates the fact that the temperatures present in the respective relative humidity classes increase from left to right. In the 40% RH measurements with directed airflow, the specific evaporation rate (${\dot{e}}_{A}$) was observed to increase rapidly from 23 °C to 27 °C, before subsequently decreasing. This trend was not observed in measurements conducted without directed airflow, which demonstrated a general decrease in the evaporation rate with increasing temperature. No recognisable trends were evident in the 70% RH measurements, with the values remaining relatively consistent. In general, the specific evaporation rates at 40% RH were considerably higher than those at 70% RH. Furthermore, no dependence of the specific evaporation rate on the saturation deficit could be identified.

We want to highlight the following findings:

- At 70% RH, all larger snails showed a higher specific evaporation rate (${\dot{e}}_{m}$ & ${\dot{e}}_{A}$) at temperatures > 30 °C than the smaller individuals.
- Among all measurements with no directed airflow the highest evaporation and specific evaporation rates were observed at 23 °C.
- With a directed airflow the highest evaporation and specific evaporation rates at 40% RH occurred at 27 °C. At 70% RH, the highest rates were observed at 27 °C in the *S* groups and at 31 °C in the *L* groups.
- In general, the recorded values at 40% RH were considerably higher than at 70% RH.
- No evidence was found to suggest that the specific evaporation rate (${\dot{e}}_{A}$) is dependent on the saturation deficit of the air.

### Specific evaporation as a response variable

The specific evaporation rate ${\dot{e}}_{m}$ was tested vs. the explanatory variables ‘average temperature’ (ϑ_*avg.*_) in °C, ‘average relative humidity’ (*RH*_*avg.*_) in % and ‘average air velocity (*v*_*avg.*_) in m × s^−1^’ with crossing explanatory variables by MLR. No multicollinearity was present in the explanatory variables (*VIF* = 1.0–3.4) and the residuals were independent (Durbin-Watson: *d* = 1.84), have constant variance (Homoscedasticity), and are normally distributed.

**Fig. 6:**
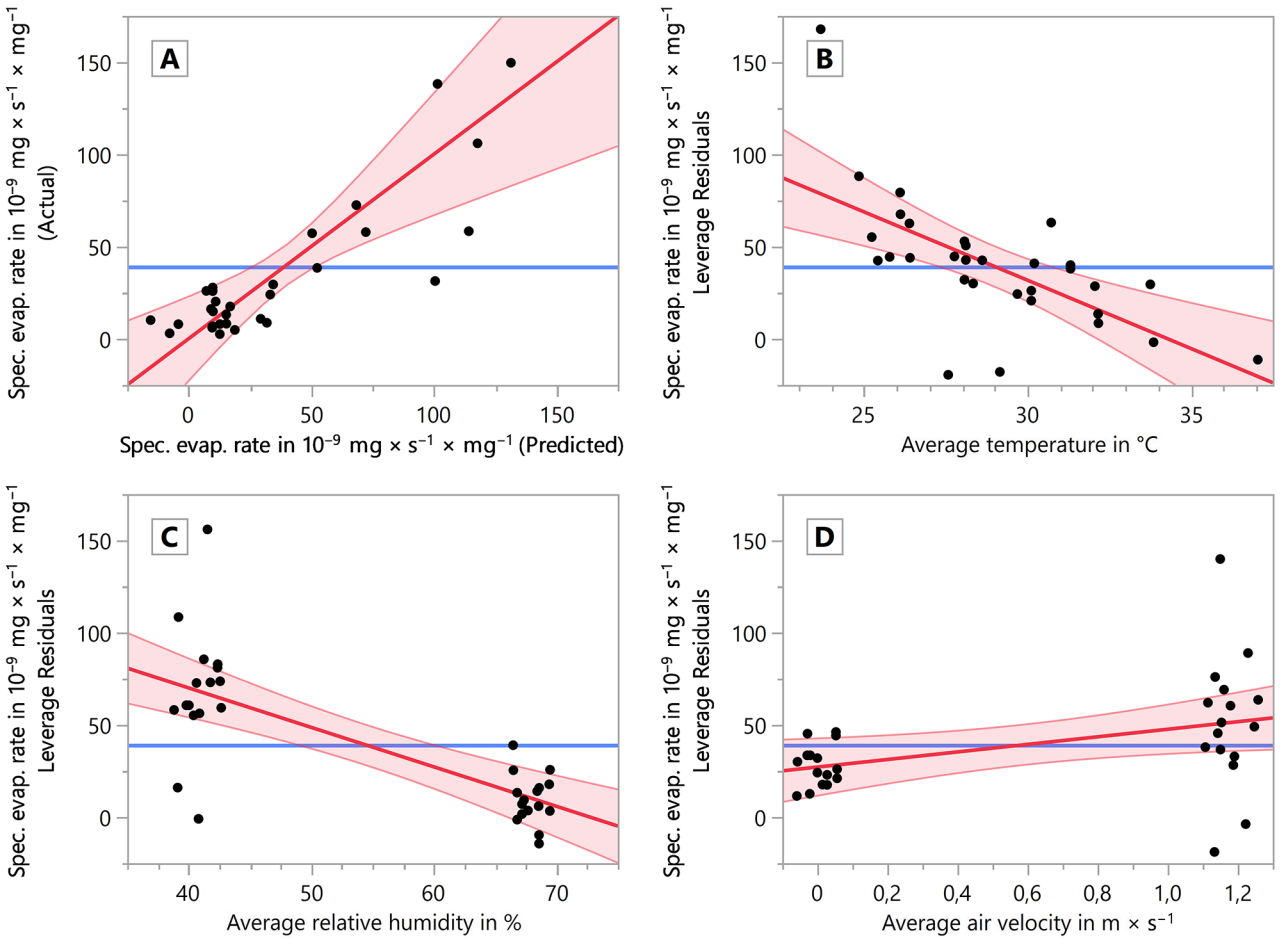
Evaporation rate as a response variable of the average temperature, average relative humidity, average air velocity, average shell-free mass and average shell aperture with crossing explanatory variables in the MLR. (**A**) Model showing predicted (abscissa) vs. actual (measured) data (ordinate), comparing the model against the null hypothesis. (**B**) Effect Leverage plot of the average temperature vs. evaporation rate. (**C**) Effect leverage plot of the average relative humidity vs. evaporation rate. (**D**) Effect leverage plot of the average air velocity vs. evaporation rate. All plots show the linear regression as a thick red line, the 95% confidence intervals as thin lighter red lines and the null hypothesis (mean of the response) as a thick blue horizontal line

In Fig. 6A the fit model of the MLR is shown displaying the model-predicted values (abscissa) vs. the measured data (ordinate). The predict expression is

4 $$\eqalign{{{\dot e}_m} = & - 207.83 - 7.4067 \times \vartheta - 2.1396 \times RH + 20.495 \times {v_{air}} \cr & + \>0.22823 \times \left( {\vartheta - 29.001} \right) \times \left( {RH - 54.419} \right) \cr & -\> 8.4544 \times \left( {{v_{air}} - 0.58932} \right) \times \left( {\vartheta - 29.001} \right) \cr & - \>1.6219 \times \left( {{v_{air}} - 0.58932} \right) \times \left( {RH - 54.419} \right)\, \cr & + \>433.76 \times {\left( {{v_{air}} - 0.58932} \right)^2} \cr} $$

with the resulting evaporation rate ${\dot{e}}_{m}$ in 10^−9^ mg × s^−1^ × mg^−1^ and the explanatory variables with the units mentioned above.

Figure 6B–D shows the leverage plots of the explanatory variables of the MLR. The *R*^2^ value of the entire model was 0.728. The parameter ‘average temperature’ (*p* = 0.0004, *F* = 16.99; Fig. 6B) and ‘average relative humidity’ (*p* < 0.0001, *F* = 29.90; Fig. 6C) yielded a highly significant (**) and the parameter ‘average air velocity’ (*p* = 0.0333, *F* = 5.097; Fig. 6D) yielded a significant (*) contribution to the model. Furthermore, the average temperature and the average relative humidity were crossed, along with the average velocity with all other explanatory variables, in the MLR. The results of the effect tests of all explanatory variables, including crossings, are shown in Table 1.

**Table 1:** Results of the effect test of explanatory variables and crossings of the multiple linear regression

Explanatory variables	*p*-value	*F*-value	Significance
average temperature	0.0004	16.99	**
average relative humidity	< 0.0001	29.90	**
average air velocity	0.0333	5.097	*
average temperature × average relative humidity	0.0152	6.828	*
average air velocity × average temperature	0.0093	8.004	**
average air velocity × average relative humidity	0.0200	6.207	*
average air velocity × average air velocity	0.0084	8.233	**

The consideration of a full-factorial interaction between all explanatory variables in the MLR did not result in a substantial improvement of the model. The model was optimised to provide the most comprehensive explanation possible, with all variables included in a significant form.

## Discussion

The present study investigated whether *T. pisana* is able to actively reduce its evaporation rate, or whether increased ambient and thus also body temperature and other boundary conditions result in an increased evaporation rate in order to avoid overheating.

Measurements in this study were taken over 24 h with inactive snails in a well hydrated state. A decrease in evaporation rate over a longer period of time was not observed as the decrease in mass (water) over time was linear. This is in accordance with the results of Petschl [31], who carried out measurements between one and 96 h. It might be that longer exposure periods lead to a decrease in the evaporation rate with time, as reported by Arad [30], or that high temperatures in combination with low relative humidity lead to a decrease after a few hours, as reported by Warburg [24].

In general, the snails, with a moist body surface, seem to feel more comfortable at higher relative humidities, which has resulted in a more dispersed pattern of the measured values of the evaporation rate, in comparison to lower relative humidities. Under the latter conditions the snails lost more water by evaporation and, thus, it may have been of greater importance for them to react to environmental conditions in order to avoid desiccation or overheating.

In the following, unless otherwise stated, the term ‘specific evaporation rate’ includes both the specific evaporation rate per shell-free mass and per shell aperture area.

### Evaporation and size

Within the given species, larger snails had a lower specific evaporation rate than smaller snails up to a certain temperature, beyond which the specific evaporation rate of larger snails was higher. This temperature varied depending on whether there was a directed airflow or not. With a directed airflow, it seems that the temperature can be higher than without an airflow until the larger snails displayed a higher specific evaporation rate (${\dot{e}}_{m}$), but the trend was evident in all measurements. This is in contrast to the findings of Arad [26] and Riddle [7]. Both stated, that the body size may play a role in resilience to dehydration since larger snails have in general a lower specific evaporation rate. A reason why Arad [26] did not see the trend could be that the temperature in his experiments was too low, especially for the snail species studied (desert snails). He also did not measure the effect of different temperatures within a species. An identical experiment with *T. pisana* was carried out by Arad in 1990 [29] and in 2001 [30]. While in 1990 the snails were smaller (lighter) than in 2001, the specific evaporation rate was lower. This also seems to be in contrast to the findings of Arad [26] and Riddle [7]. However, there may be many reasons for the variation, such as different populations and therefore different preconditions. An explanation for the trend found in the present study could be related to the fact that larger snails have a larger water reservoir and can therefore afford to evaporate more water at high temperatures in favour of cooling and are not directly threatened by desiccation. Alternatively, a simple geometric explanation may apply: with increasing size (= diameter), the volume (∝ mass) increases to power of three and the evaporation surface (= shell aperture area) only to the power of two. Therefore, larger snails are not able to evaporate as much as smaller snails can in relation to their mass.

The evaluation of the evaporation rate with regard to shell-free mass and shell aperture area did not change the interpretation of the data. Both specific evaporation rates did show, more or less, the same picture. It can therefore be assumed that the values of the results are not due to the method of data calculation, but in fact to the response of the snails.

### Environmental impact

As stated in the literature, rising temperature was identified as a factor that increases evaporation rates [24, 27, 31]. This could not be universally confirmed in this study, as the given results strongly indicate that the snails were actively responding to the environmental conditions. No clear trend could be identified; rather, a certain temperature threshold was found to induce a reaction in the snails, resulting in a reduction in evaporation, depending on different other environmental conditions as relative humidity and air velocity. The adaptive behaviours triggered by high temperatures – such as induction of aestivation [15, 35], seeking shelter in subterrestrial microhabitats [14, 36], and daytime inactivity – are well-documented strategies to prevent excessive water loss (summarised by Schweizer et al. [17]). Thus, it seems reasonable that an increase in temperature by itself does not necessarily lead to an increased evaporation.

With a directed airflow, the maximum values of the (specific) evaporation rates were considerably higher than in the measurements conducted without. With regard to relative humidity, the following patterns emerged: at 40% RH, all values of the (specific) evaporation with a directed airflow were higher except the values at 35 °C in group *L*. At 70% RH, the values with a directed airflow at 23 °C and 35 °C were lower, at 31 °C the values were higher and at 27 °C the values in group *L* were lower, and in group *S* they were higher. This provides evidence to support the hypothesis that the snails are capable of regulating evaporation, when necessary, particularly in consideration of the necessity for this adaptation at low relative humidity.

Generally, the (specific) evaporation rate was found to be considerably higher at 40% RH than at 70% RH. Given that humid air with a higher relative humidity can absorb relatively less water, this result appears to be in accordance with the expected outcome. Nevertheless, in the existing literature, the relative air humidity was neglected in almost all studies, as it was simply not measured, or several parameters had changed. In the studies of Warburg [24] the relative humidity increased with increasing temperature as he had intended to establish a constant saturation deficit over the temperature range. From a physical perspective, this appears to be the optimal approach, as only a single parameter was changed in his experiments: the temperature. As the pressure gradient (saturation deficit) between the aqueous phase within the snail and the surrounding air is the basis for evaporation, this seems to be the most significant factor to consider. A different perspective is obtained by plotting the results of this study against the saturation deficit (Fig. 5). A higher saturation deficit did indeed lead to a higher evaporation rate, considering the data points measured at the same temperature, but it was also evident that a higher relative humidity (cluster on the bottom left-hand side of Fig. 5) has led to a lower evaporation rate.

### Regression analysis

The specific evaporation rate as a function of multiple explanatory variables showed that 72.8% of all variation in the specific evaporation rate could be explained by variation in temperature, relative humidity and air velocity. The impact of all three explanatory variables has been verified with regard to the slope of the linear regression and the *p* values of the linear regression model. The leverage plots of the explanatory variables (Fig. 6B–D), indicated the effect of the variable in question, given that all other effects were already considered in the model. No influential points or multicollinearity were evident.

### Alignment with existing research

As shown in the previous sections, the (specific) evaporation rate is dependent upon many variables, and the snail itself is capable of actively reducing the evaporation rate if necessary. This therefore makes it difficult to compare our results to published data on (specific) evaporation rates between different snail species and even within a single species. Nevertheless, the intra- and interspecific variation of this parameter is of great interest for the assessment of biodiversity and, thus, the average (specific) evaporation rates of different snail species in the literature are summarised in Table 2.

**Table 2:** Evaporation of different snail species in literature

Species	Avg. Evaporation Rate*^1^ in 10^−6^ mg × s^−1^	Avg. Spec. Evaporation Rate*^2^ in 10^−9^ mg × s^−1^ × mg^−1^	Conditions*^3^	Reference
*Theba pisana*	63.0 (8.20) / 3.49 (0.399)	98.2 / 4.50	23 °C, inact., RH: 40% / 70%, *v*_*air*_ = 1 m × s^−1^	present study
	134 (17.6) / 7.73 (0.94)	189 / 9.77	27 °C, inact., RH: 40% / 70%, *v*_*air*_ = 1 m × s^−1^	
	46.9 (5.45) / 11.7 (1.34)	68.6 / 11.2	31 °C, inact., RH: 40% / 70%, *v*_*air*_ = 1 m × s^−1^	
	18.5 (2.35) / 7.41 (0.964)	24.8 / 9.03	35 °C, inact., RH: 40% / 70%, *v*_*air*_ = 1 m × s^−1^	
	47.7 (6.25) / 14.7 (1.92)	65.2 / 21.1	23 °C, inact., RH: 40% / 70%, *v*_*air*_ = 0 m × s^−1^	
	39.5 (4.47) / 9.83 (1.21)	47.8 / 10.8	27 °C, inact., RH: 40% / 70%, *v*_*air*_ = 0 m × s^−1^	
	19.2 (2.38) / 5.03 (0.635)	23.8 / 5.27	31 °C, inact., RH: 40% / 70%, *v*_*air*_ = 0 m × s^−1^	
	20.8 (2.65) / 8.95 (1.15)	26.8 / 10.6	35 °C, inact., RH: 40% / 70%, *v*_*air*_ = 0 m × s^−1^	
*Pleuroxia sp. &* *Pupoides adelaidae*	2.14		20 °C, RH: 49.2%, dorm., w.e., 8 h	Warburg [24]
	2.57		25 °C, RH: 62.1%, dorm., w.e., 8 h	
	3.32		30 °C, RH: 71.7%, dorm., w.e., 8 h	
	5.82 / 4.20		35 °C, RH: dry air / 78.3%, dorm., w.e., 8 h	
	7.10		40 °C, RH: dry air, dorm., w.e., 8 h	
*Cepaea nemoralis*	199 / 2264	92.5 / 1011	28 °C, RH: 60–70%, inact. / act., #	Cameron [19]
*Cepaea hortensis*	181 / 1457	121 / 990		
*Arianta arbustorum*	242 / 2358	169 / 1153		
*Sphincterochila zonata*	87.0	39.8	25 °C, RH: NA, 504 h, #	Arad et al. [25]
*Sphincterochila prophtarum*	79.3	114		
*Sphincterochila fimbriata*	73.7	78.9		
*Sphincterochila aharonii*	61.4	126		
*Sphincterochila cariosa*	59.5	100		
*Trochoidea simulata*	47.2	39.6	25 °C, RH: NA, 504 h, #	Arad [29]
*Xeropicta vestalis*	11.6	23.4		
*Monacha haifaensis*	20.3	81.1		
*Theba pisana*	25.4	53.5		
*Eremina desertorum*	120	48.1	25 °C, RH: dry air, #	Arad [26]
*Euchondrus desertorum*	9	75.2		
*Euchondrus albulus*	5.4	104		
*Chrisataria genezarethana*	59.6 / 13.1	1675 / 367	25 °C, RH: NA, 24 h / 168 h, #	Arad et al. [28]
*Rupestrella rhodia*	75.0 / 22.5	1443 / 432	25 °C, RH: NA, 24 h / 168 h, #	
*Levantina caesareana*	5470 / 2004	591 / 217	25 °C, RH: NA, 24 h / 168 h, w.e., #	
*Theba pisana*	58.5	63.5	25 °C, RH: NA, 504 h, #	Arad [30]
	45.0	54.9	25 °C, RH: NA, 840 h, #	
*Xeropicta derbentina*	(178 / 31.6 / 18.8 / 13.4)		20 °C, RH: NA, inact., 1 h / 8 h / 24 h / 48 h, #	Petschl [31]
	(61.4 / 44.0 / 41.6 / 38.3)		35 °C, RH: NA, inact., 1 h / 8 h / 24 h / 76 h, #	
	(167 / 47.8 / 43.4)		40 °C, RH: NA, inact., 1 h / 8 h / 24 h, #	
	(507 / 139)		42 °C, RH: NA, inact., 1 h / 8 h, #	
	(999 / 351)		45 °C, RH: NA, inact., 1 h / 8 h, #	

*^1^ Values in parentheses are given in 10^−6^% × s^−1^ (loss refers to the initial shell-free mass)

*^2^ Per shell-free mass

*^3^ Activity: active (act.), inactive (inact.) & dormant (dorm.); RH specifications: No data available (NA), values in % if given; The designation “w.e.” indicates that the snails built an epiphragma in the experiment; Values in hours (h) are the duration of the experiment if given; Values of the reference calculated with given shell-free mass or duration of the experiment are indicated with an # “calculated”

A comparison of the average evaporation rates found in this study with those of Warburg [24] shows his values to be much lower. Considering the size (diameter) of the species investigated by him (*Pleuroxia* sp.: 11–17 mm; *Pupoides adelaidae*, former *Themapupa adelaidae*: 6–7 mm) compared to *T. pisana*: 12–25 mm, this seems reasonable as the size, per se, is relevant for evaporation. *Pleuroxia* sp. and *T. pisana* both belong to the Helicoidea and, therefore, the shell shapes and proportions, apart from the size, are quite similar. Given the volume, *T. pisana* should have an evaporation rate 1.3 to 3.2 times higher than *Pleuroxia* sp., based on a sphere (∝ diameter^3^). In fact, values are more than 22 time higher (compared 23 °C & 40% RH and 20 °C & 49.2% RH) than the reported ones at lower temperatures. However, the factor decreased to approximately 3.5 (compared 35 °C & 40% RH and 35 °C & dry air) at high temperatures. One major reason for this difference could be the general dormancy of the investigated snails of Warburg [24] which is at best comparable to the inactivity of our individuals at higher temperatures. *Cepaea nemoralis*,* Cepaea hortensis* and *Arianta arbustorum* investigated by Cameron [19] were similar in size to *T. pisana*, and all four species also belong to the Helicoidea. The specific evaporation rate of *T. pisana* was 8.6–15.6 times lower than that of the other three species under similar conditions (27 °C & 70% RH and 28 °C & 60–70% RH). One reason could be the different habitat (woodlands, dunes and open biotopes) as stated by Arad et al. [28] compared with rock-dwelling snails, and by Asami [27] who attributed the differences in his findings on the evaporation rate of three species to the different physiological or (and) behavioural responses to environmental desiccation. The individuals investigated by Arad et al. [28] that did not form a epiphragma had a 22–155 time higher specific evaporation rate than *T. pisana* under similar conditions. Arad et al. [25] reported that Mediterranean-type water economy species, like *Sphincterochila prophetarum*,* Sphincterochila aharonii* and *Sphincterochila cariosa* require 3–4 days to initiate water-preserving mechanisms. This should also include *T. pisana*, as its habitat also lies in the Mediterranean region, but, nevertheless, the specific evaporation rates recorded in the present study were 1.5–11.7 times lower than those of the aforementioned species under similar conditions [29, 30]. Comparing the evaporation rate (based upon the initial shell-free mass) of *X. derbentina*, a species competing with *T. pisana* in the same habitat, that was studied by Petschl [31], indicates that *T. pisana* seems to be better adapted to its habitat, with an evaporation rate being 3.0–9.8 times lower at lower temperatures and 2.0–2.3 times lower at higher temperatures. Desert species such as *Sphincterochila zonata* [25], *Trochoidea simulata* [29] or *Eremina desertorum* [37] have a slightly, i.e. 1.0–1.6 times, lower specific evaporation rate than *T. pisana*. This indicates an adaptation of the studied species *T. pisana* to dry and hot habitats, at least with respect to appropriate water retention. Schmidt-Nielsen et al. [38] has measured a mass loss of 0.66 mg × d^−1^ in the desert snail *Sphincterochila zonata (*formerly *boissieri)*. The measurements were taken in the field under natural conditions with inactive snails. Compared to these results, the evaporation rate of *T. pisana* was 2.4–6.1 times higher under near-natural conditions (31 °C and 35 °C & 40% RH), assuming that both species are of similar size. In fact, *S. zonata* is a slightly smaller, but there was no information about the size of the individuals used by them, so the factor between the results could be smaller.

The comparison of the data should be treated with caution, as some of the snail species inhabit different (micro-) habitats and have different physiological adaptations to high temperatures. Thermal tolerance, the temperature at which 50% die, or lethal temperature could be indicators of this. The thermal tolerance of *T. pisana* ranges from 50.7 to 52.6 °C, depending on the region of origin [39]. The lethal temperature for this species is 55 °C over a period of three to four hours [38, 40].

Based on the assumption of Machin [6] that a snail can survive a loss of at least 35% of its water content, the survival time of *T. pisana* under the experimental conditions can be calculated to 18 days in the worst case (27 °C, 40% RH, *v*_*air*_ ≈ 1.2 m × s^−1^) and, in the best case, to over 4 years (Group *S*, 31 °C, 70% RH, *v*_*air*_ ≈ 0 m × s^−1^) for the smaller snails, and 27 days in the worst case (27 °C, 40% RH, *v*_*air*_ ≈ 1.2 m × s^−1^) and in the best case to over 3.5 years (23 °C, 70% RH, *v*_*air*_ ≈ 1.2 m × s^−1^) for larger snails. Such theoretically calculated values are of course prevented by the far shorter lifetime of this species. This finding again shows that smaller and larger snails react differently to environmental conditions, but also shows that smaller snails are not clearly disadvantaged during desiccation. The duration of our measurements was only 24 h and therefore the previous statements should be treated with caution, as it is not possible to draw any conclusions about the behaviour of the snails during longer periods of drought where the trade-off between water conservation and evaporative cooling potentially changes.

We are well aware that the measured data are restricted, as it is an artificial experiment carried out in the laboratory and can therefore only be compared with field measurements to a limited extent. Furthermore, it was assumed that the snails would all behave in the same way, but this is not the case. Nevertheless, data from a laboratory experiment are of great interest, as the number of variables can be significantly reduced and controlled compared to field measurements.

In the context of *Theba pisana*’s ecology, the observed capacity to regulate water loss and preserve internal water likely represents a key adaptation to its arid and seasonally dry habitats. As a Mediterranean land snail often exposed to high temperatures and prolonged periods of low humidity, *T. pisana* faces strong selective pressures to minimise desiccation risk. Mechanisms such as reduced cutaneous permeability, behavioural estivation, and mucus conservation can thus be interpreted as critical survival strategies during drought or inactivity. The physiological trends observed in this study may enhance survival during the hot, dry summer months when metabolic activity is reduced and water availability is limited. From an evolutionary perspective, such traits likely reflect adaptation to xeric environments and may have evolved under selection for desiccation resistance. Similar water-conservation strategies are seen in other pulmonate gastropods occupying dry habitats, suggesting either convergent evolution or conservation of ancestral traits in snails’ habitats adapted to terrestrial life.

## Conclusion

The study focused on how evaporation is affected by environmental conditions and whether *T. pisana* is able to actively reduce its evaporation rate, or whether increased environmental and thus also body temperature and other boundary conditions lead to increased evaporation in order to avoid overheating. In the given temperature range, the snails tended to actively reduce their evaporation rate which can be seen as a water preservation strategy. This observation leads to the conclusion that at higher temperatures (35 °C), the snails do not increase their evaporation rate in order to avoid overheating.

This study has led to the following findings that will be of importance for future thermodynamic models on land snails:

1. Multiple regression modelling shows that the specific evaporation rate can be well described (72.8%) by the parameters temperature, relative humidity and air velocity.
2. The results indicate that the response of *T. pisana* to environmental conditions affects evaporation. No clear trend could be found to confirm that an increase in temperature leads to an increase in evaporation rates; rather, it was found that a certain threshold temperature triggers a response in the snails that leads to a reduction in evaporation.
3. Larger snails have a lower specific evaporation rate than smaller snails up to a certain temperature, beyond which the specific evaporation rate of larger snails is higher, depending on the air velocity.
4. Evaporation rates are generally lower at higher relative humidities.
5. The shell aperture area (in mm²) can be calculated by multiplying the diameter (in mm) squared by the factor 0.1805.

With the measurements obtained in the present study and the accepted correlations with abiotic parameters, as well as the data collected by Zimmermann et al. [33] on the modulation of metabolism, and generally known physical correlations, the prerequisites are given for the construction of a complete thermodynamic model for this species.

## Electronic supplementary material

Below is the link to the electronic supplementary material.

Supplementary Material 1

## Data Availability

No datasets were generated or analysed during the current study.
