# Supplementary material for: Water evaporation as a function of temperature, humidity, air velocity and body size in inactive terrestrial pulmonate Theba pisana
Source: BMC Zool. 2025 Jul 7;10:13. doi: 10.1186/s40850-025-00236-0 (PMC12232781; doi:10.1186/s40850-025-00236-0)
Supplement: Supplementary file 1 — Supplementary Material 1 [file 40850_2025_236_MOESM1_ESM.docx]

**Table S1**. Data set comprising all measurements conducted in this study including diameter, shell-free mass, oxygen consumption of inactive and measurable active snails and the activity of the snails determined by cam or by increase of the relative humidity.

| **Measurement** | **Avg. Evaporation Rate** | **Avg. Shell-free Mass** | **Avg. Diameter ± SD** | **Avg. Shell Aperture** | **Temperature ± SD** | **Rel. Humidity ± SD** | **Air Velocity ± SD** |
| --- | --- | --- | --- | --- | --- | --- | --- |
|  | in 10^−6^ mg × s^−1^ | in mg | in mm | in mm^2^ | in °C | in % | in m/s |
| M13 | 3.472 | 577.6 | 13.58 ± 0.18 | 33.27 | 23.00 ± 0.06 | 67.55 ± 0.37 | 1.07 ± 0.24 |
| M16 | 3.508 | 1172.0 | 17.05 ± 0.16 | 52.48 | 23.00 ± 0.06 | 67.57 ± 0.42 | 1.03 ± 0.23 |
| M15 | 62.999 | 456.1 | 12.78 ± 0.16 | 29.50 | 23.01 ± 0.02 | 41.64 ± 0.78 | 1.03 ± 0.25 |
| M14 | 62.940 | 1079.2 | 16.59 ± 0.22 | 49.66 | 23.01 ± 0.02 | 41.52 ± 0.77 | 1.05 ± 0.24 |
| M08 | 5.465 | 504.3 | 12.88 ± 0.35 | 29.96 | 27.00 ± 0.05 | 67.93 ± 0.28 | 1.18 ± 0.27 |
| M07 | 9.992 | 1146.8 | 17.03 ± 0.17 | 52.34 | 27.00 ± 0.04 | 67.93 ± 0.20 | 1.19 ± 0.27 |
| M06 | 115.992 | 507.7 | 12.99 ± 0.19 | 30.45 | 27.00 ± 0.06 | 41.84 ± 1.85 | 1.21 ± 0.27 |
| M05 | 151.248 | 1011.0 | 16.45 ± 0.24 | 48.87 | 27.00 ± 0.02 | 41.60 ± 1.01 | 1.20 ± 0.27 |
| M04 | 2.773 | 574.4 | 13.52 ± 0.24 | 32.99 | 31.00 ± 0.01 | 67.63 ± 0.07 | 1.23 ± 0.29 |
| M03 | 20.685 | 1179.4 | 17.04 ± 0.25 | 52.42 | 31.00 ± 0.01 | 67.64 ± 0.08 | 1.23 ± 0.29 |
| M02 | 57.282 | 540.7 | 13.09 ± 0.36 | 30.94 | 31.00 ± 0.02 | 39.26 ± 0.06 | 1.27 ± 0.30 |
| M01 | 36.437 | 1163.2 | 17.02 ± 0.19 | 52.32 | 31.00 ± 0.02 | 39.26 ± 0.11 | 1.24 ± 0.29 |
| M11 | 3.793 | 478.5 | 12.84 ± 0.20 | 29.77 | 35.00 ± 0.01 | 69.17 ± 0.06 | 1.27 ± 0.27 |
| M12 | 11.034 | 1088.7 | 16.76 ± 0.27 | 50.68 | 35.00 ± 0.01 | 69.17 ± 0.05 | 1.25 ± 0.26 |
| M10 | 16.438 | 558.2 | 13.49 ± 0.23 | 32.84 | 35.00 ± 0.02 | 39.23 ± 0.02 | 1.24 ± 0.27 |
| M09 | 20.491 | 1014.6 | 16.39 ± 0.24 | 48.47 | 35.00 ± 0.02 | 39.23 ± 0.01 | 1.19 ± 0.27 |
| M30 | 12.475 | 480.5 | 12.77 ± 0.16 | 29.46 | 23.00 ± 0.06 | 67.93 ± 0.95 | 0 |
| M29 | 16.901 | 1046.6 | 16.80 ± 0.11 | 50.93 | 23.00 ± 0.05 | 67.19 ± 1.24 | 0 |
| M28 | 35.200 | 485.7 | 13.08 ± 0.08 | 30.89 | 23.01 ± 0.06 | 43.97 ± 3.32 | 0 |
| M19 | 59.304 | 1025.3 | 16.37 ± 0.15 | 48.39 | 23.01 ± 0.06 | 42.45 ± 2.89 | 0 |
| M20 | 3.793 | 558.7 | 13.36 ± 0.15 | 32.22 | 27.00 ± 0.05 | 67.99 ± 0.86 | 0 |
| M21 | 15.871 | 1068.4 | 16.66 ± 0.15 | 50.12 | 27.00 ± 0.03 | 67.88 ± 0.84 | 0 |
| M18 | 33.987 | 594.2 | 13.71 ± 0.13 | 33.94 | 27.00 ± 0.05 | 43.27 ± 2.08 | 0 |
| M17 | 45.100 | 1173.4 | 17.09 ± 0.16 | 52.70 | 27.00 ± 0.03 | 42.00 ± 2.27 | 0 |
| M24 | 1.217 | 475.7 | 12.95 ± 0.10 | 30.25 | 31.00 ± 0.01 | 67.63 ± 0.36 | 0 |
| M25 | 8.843 | 1108.0 | 17.06 ± 0.18 | 52.53 | 31.00 ± 0.01 | 67.63 ± 0.36 | 0 |
| M22 | 13.198 | 550.5 | 13.64 ± 0.12 | 33.59 | 31.00 ± 0.02 | 39.37 ± 0.37 | 0 |
| M23 | 25.229 | 1064.3 | 16.93 ± 0.15 | 51.75 | 31.00 ± 0.02 | 39.15 ± 0.40 | 0 |
| M32 | 4.052 | 493.8 | 12.98 ± 0.14 | 30.40 | 35.00 ± 0.01 | 69.35 ± 0.31 | 0 |
| M31 | 13.855 | 1058.6 | 16.96 ± 0.14 | 51.90 | 35.00 ± 0.01 | 68.97 ± 0.44 | 0 |
| M27 | 15.150 | 545.1 | 13.71 ± 0.12 | 33.95 | 35.00 ± 0.02 | 39.24 ± 0.22 | 0 |
| M26 | 26.534 | 1026.3 | 16.28 ± 0.13 | 47.86 | 35.00 ± 0.02 | 39.22 ± 0.25 | 0 |

|  |
| --- |
|  |
